# The influence of cross-sectoral treatment models on patients with mental disorders in Germany: study protocol of a nationwide long-term evaluation study (EVA64)

**DOI:** 10.1186/s12888-018-1721-z

**Published:** 2018-05-18

**Authors:** Anne Neumann, Enno Swart, Dennis Häckl, Roman Kliemt, Stefanie March, Denise Küster, Katrin Arnold, Thomas Petzold, Fabian Baum, Martin Seifert, Jessika Weiß, Andrea Pfennig, Jochen Schmitt

**Affiliations:** 1 Center of Evidence-based Health Care, Medizinische Fakultät Carl Gustav Carus, University Hospital Carl Gustav Carus, Technische Universität Dresden, Fetscherstraße 74, 01307 Dresden, Germany; 2 Institute of Social Medicine and Health Economics, Medical Faculty, Otto-von-Guericke- University Magdeburg, Magdeburg, Germany; 3 WIG2 Scientific Institute for Health Economics and Health System Research Leipzig, Leipzig, Germany; 4 Clinic for Child and Adolescence Psychiatry and Psychotherapy, Carl Gustav Carus University Hospital, Technische Universität Dresden, Dresden, Germany; 5 Department of Psychiatry and Psychotherapy, Carl Gustav Carus University Hospital, Technische Universität Dresden, Dresden, Germany

**Keywords:** Claims data, Psychiatric health care, Effectiveness and cost-effectiveness, Routine data, Statutory health insurance, Inpatient and outpatient treatment, Setting approach, Health care system, Health services research

## Abstract

**Background:**

Close, continuous and efficient collaboration between different professions and sectors of care is necessary to provide patient-centered care for individuals with mental disorders. The lack of structured collaboration between in- and outpatient care constitutes a limitation of the German health care system. Since 2012, a new law in Germany (§64b Social code book (SGB) V) has enabled the establishment of cross-sectoral and patient-centered treatment models in psychiatry. Such model projects follow a capitation budget, i.e. a total per patient budget of inpatient and outpatient care in psychiatric clinics. Providers are able to choose the treatment form and adapt the treatment to the needs of the patients. The present study (EVA64) will investigate the effectiveness, costs and efficiency of almost all model projects established in Germany between 2013 and 2016.

**Methods/design:**

A health insurance data-based controlled cohort study is used. Data from up to 89 statutory health insurance (SHI) funds, i.e. 79% of all SHI funds in Germany (May 2017), on inpatient and outpatient care, pharmaceutical and non-pharmaceutical treatments and sick leave for a period of 7 years will be analyzed. All patients insured by any of the participating SHI funds and treated in one of the model hospitals for any of 16 pre-defined mental disorders will be compared with patients in routine care. Sick leave (primary outcome), utilization of inpatient care (primary outcome), utilization of outpatient care, continuity of contacts in (psychiatric) care, physician and hospital hopping, re-admission rate, comorbidity, mortality, disease progression, and guideline adherence will be analyzed. Cost and effectivity of model and routine care will be estimated using cost-effectiveness analyses. Up to 10 control hospitals for each of the 18 model hospitals will be selected according to a pre-defined algorithm.

**Discussion:**

The evaluation of complex interventions is an important main task of health services research and constitutes the basis of evidence-guided advancement in health care. The study will yield important new evidence to guide the future provision of routine care for mentally ill patients in Germany and possibly beyond.

**Trial registration:**

This study was registered in the database “Health Services Research Germany” (trial number: VVfD_EVA64_15_003713).

## Background

Mental disorders are complex and characterized by a long duration until adequate diagnosis and treatment [1]. The German Bundestag Study Commission on Psychiatry urged, already in the 1970ies, for new models of care aiming at, e.g., preference of outpatient over inpatient care (where possible), the equality of mentally and somatically ill patients and a regionalized health care [2]. A continuous, close and efficient collaboration between different professions and sectors of care is necessary to provide patient-centered care for patients with mental health problems [3, 4]. Insufficient interfaces between different health care sectors are a fundamental problem in the current German healthcare system, but particularly of concern in psychiatric care [5]. Insufficient trans-sectoral interfaces concern the transition from inpatient to outpatient care, the joint care of patients involving several specialists and the transition from rehabilitation to the first labor market [6, 7].

In addition, the financing of the German psychiatric health care system is currently fragmented constituting another barrier towards efficient collaboration across sectors [8]. The current remuneration of care for mentally ill patients has been suspected to lead to misdirected incentives for inappropriate or inappropriately long inpatient care [9] and more resource intensive treatment [10].

Inadequate information about available treatment programs, inadequate collaboration between sectors and health care professions, misleading communication and long waiting periods lead to difficulties in care and, therefore, often to deterioration of the individual wellbeing of those affected [4, 11, 12]. 75% of most mental disorders manifest between age seven and 24 [1]; and those disorders often persist over many years [13]. Therefore, a sound collaboration between care of children and adolescents with adult care including a joint care during transition into adulthood is vital, though often not processed adequately in Germany.

About 87% of the German population (i.e. 71.9 million people) is insured through statutory health insurance (SHI) funds, and about 10% is covered by private health insurance (PHI) funds [14–16]. SHI funds cover all employees with a gross income of up to a contribution assessment ceiling (EUR 3825 per month or EUR 45,900 per year as of 2012) and their non-working family members (spouses and children) [14]. Individuals whose income is above the contribution assessment ceiling can voluntarily enroll in SHIs or switch to PHIs [14].

Several health care models that aim to change the incentives in the current system, to improve medical care of mentally ill patients and to arrive at a more rational use of resources have been investigated [8, 17]. Since 2012, a new law (§64b Social Code Book (SGB) V) has enabled the establishment of models that focus on cross-sectoral and patient-centered health care for mentally ill patients in Germany. For this, SHI funds can contract with hospitals and jointly establish the new structure according to §64b SGB V (model contracts). The common characteristic of all model contracts / model projects is a total budget of inpatient care and hospital-based psychiatric outpatient clinic (capitation principle).

According to the capitation principle, a lump sum is allocated to the model hospital each year of contract. If the patient has to be treated more than once in a year, the patient is only counted once but the treatment has to be paid by the model hospital [18]. The model hospital has to cover all of its expenses with the contracted lump sum, but is free to offer all forms of treatments, including inpatient, outpatient or home treatment. The model hospital can construct models of care that suit the region and meet the community members’ needs [19]. International and national results indicate that systems under capitation principle can be as effective as routine care [20], more effective in the short-term [21, 22] or even worse compared to routine care [23]. Some projects that follow the capitation principle in psychiatric care have been established in Germany (most on the basis of integrated care using §140a SGB V); however, basic information about construct and results of the projects need to be evaluated on a common basis [24]. For a shift from model projects to routine psychiatric care in Germany, a common structure, such as §64b SGB V, and a scientific common evaluation, such as the here described study, are necessary [3].

The aims of the projects according to §64b SGB V include the establishment of transparent processes and a reduction of disincentives for a cost-effective use of available resources. In specific terms, the aims of the model projects are:

(A) The implementation and advancement of an optimized patient care through cross-sectoral treatment,
(B) The enforcement of outpatient treatment options with consecutively improved adaptation of duration and intensity of treatment to the individual treatment needs of mentally ill patients,
(C) A continuous treatment and stabilization of patients under consideration of their social and occupational environment,
(D) The improvement of acceptance of patient-oriented psychiatric, psychotherapeutic and socio-therapeutic interventions,
(E) The establishment of transparency,
(F) A more cost-effective use of available resources in the health care of patients with mental disorders.

The supplementary law (§65 SGB V) further requires evaluation of all of those models.

This manuscript describes the study design of the nationwide evaluation of model projects according to §64b SGB V in Germany using data from statutory health insurance (SHI) funds (EVA64). The scientific use of claims data from SHI funds for the evaluation of new health care concepts has been established during the last years [25] including analysis and reporting standards [26, 27].

## Methods/design

### Study population / inclusion criteria model hospitals

A health insurance data-based controlled cohort study is conducted. All patients insured by any of the 89 participating SHI funds, i.e. 79% of all SHI funds in Germany (dated: May 2017 [28]), and treated in one of the model hospitals due to any of the 16 pre-defined mental disorders (Table 1) within the first 4 years after initiation of a model contract and with a minimum follow-up time of 1 year will be included. All 16 mental disorders represent about 80–85% of all psychiatric cases treated in hospitals [29]. A patient will only be included in the analysis if his or her SHI fund has a model contract with the model hospital and takes part in the evaluation. Further, patients of comparable control hospitals will be included as control patients (more information on the selection of control hospitals below). Anonymous patient data from the SHI funds will be extracted. Due to the assessment of anonymous data, it is not necessary to explicitly inform about the study and informed consent is not obtained. All participating SHI funds had examined the accordance with the data protection law.

**Table 1:** Inclusion criteria, diagnoses, International Classification of Disease, 10th revision (ICD-10)

ICD-10	Diagnosis
F00	Dementia
F01	Vascular dementia
F02	Dementia in other diseases classified elsewhere
F03	Unspecified dementia
F07	Personality and behavioral disorders due to brain disease, damage and dysfunction
F10	Mental and behavioral disorders due to use of alcohol
F20-F29	Schizophrenia, schizotypal and delusional disorders
F30-F39	Mood (affective) disorders
F43	Reaction to severe stress, and adjustment disorders
F45	Somatoform disorders
F40-F48	Neurotic, stress-related and somatoform disorders
F50	Eating disorders
F60.31	Specific personality disorders of type borderline
F70-F79	Mental retardation
F84	Pervasive developmental disorders
F90-F98	Behavioral and emotional disorders with onset occurring in childhood and adolescence

### Matching

Two stages of matching will be conducted. First, control hospitals were allocated to each model hospital. Second, patients will be matched between model and control hospitals.

### Control hospitals

The selection of comparable control hospitals was based on a pre-defined algorithm using data from structured quality reports according to §136b SGB V and matched data from the Federal Institute for Research on Building, Urban Affairs and Spatial Development (BBSR) [30], which included sociodemographic and socioeconomic data on the level of administrative districts (LANDKREISE). Since 2005, all hospitals in Germany are obliged to publish selected structural data of their hospital in structured quality reports. These data are collected in a database and available for research and the public [31]. A priori defined knock-out criteria (e.g. same region, institutionalized structures (specialist departments and psychiatric outpatients department (PIA))), criteria based on patients (i.e. number of cases per diagnosis) (weighting 50%), structural features of hospitals (e.g. number of beds or number of personnel) (25%) and regional factors (e.g. unemployment rate, household income) (25%) were used to identify structurally comparable control hospitals for each model hospital. Further details on the selection of the control hospitals based on routine data can be found elsewhere [32]. Up to 10 control hospitals for each model hospital were selected (with a minimum of eight clinics for departments of general psychiatry and a minimum of three clinics for departments of child and adolescence psychiatry).

To reach higher comparability between model and control hospitals, only the first five of the 10 identified control hospitals will be selected for analysis. If the five best matched control hospitals do not provide at least threefold the number of patients compared to the model hospital, the next best fitting control hospitals will be also selected step by step until the total number of patients from control hospitals is at least threefold as high as the number of patients in the corresponding model hospital. The remaining control hospitals might be selected later in the study if, for example, chosen control hospitals merge, close or turn into model hospitals.

### Patient matching

Second, patients will be matched exactly according to year of study inclusion, hospital-known vs. hospital-new patient (hospital-new  = no contact to psychiatric ward or PIA in the corresponding model or control hospital in the 2 years prior to study inclusion), diagnoses of mental disorders at study inclusion and with propensity score matching (nearest neighbor, caliper = 0.25 standard deviation, without replacement) according to age and sex at study inclusion and health care utilization before study inclusion. Each patient from model hospitals will be paired with one best matched patient from a control hospital, considering the match of the hospital and the match of the patient characteristics.

### Length of observation period

**Fig. 1:**
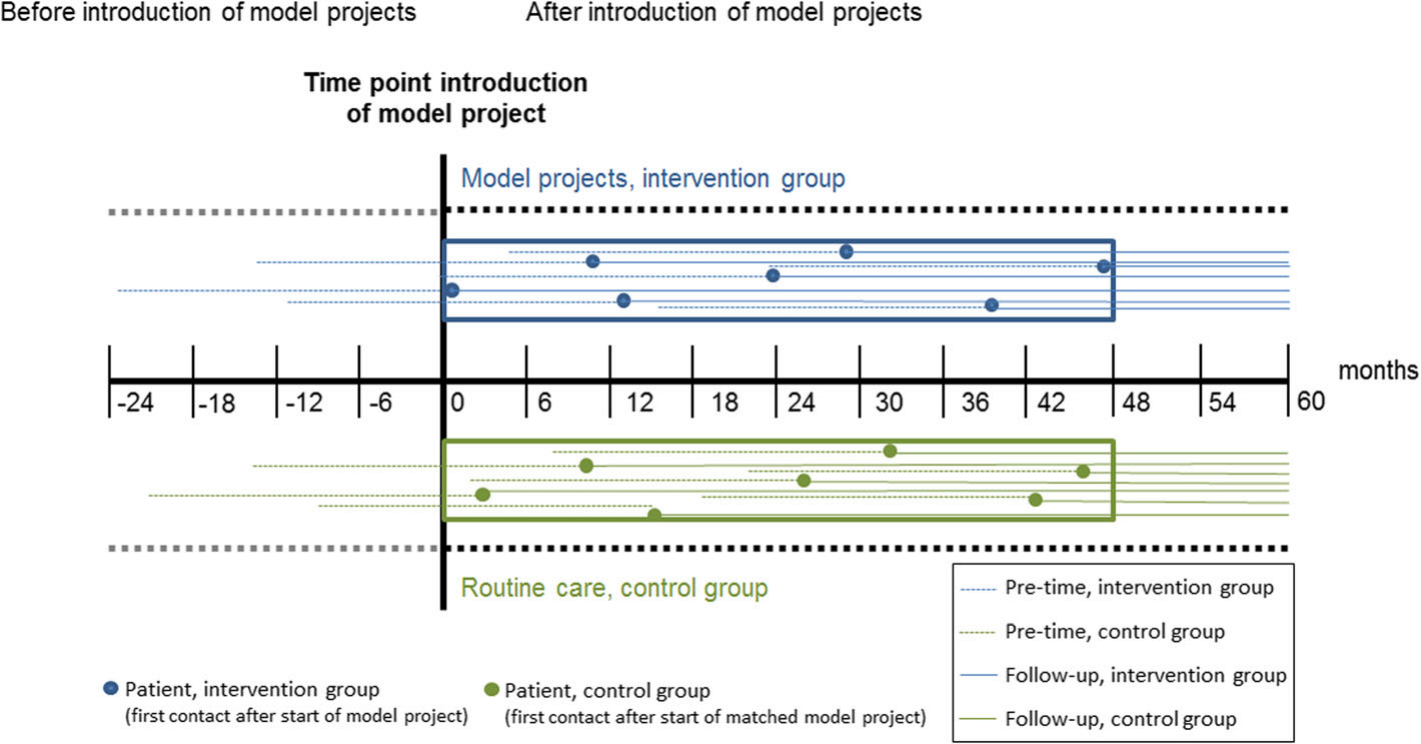
Overview study design

All patients who entered a model or control hospital within the first 4 years after start of a model contract will be included in the analysis and followed until the end of year five since model establishment. In addition, to estimate the effect of health care utilization before model start, data of 2 years before study entrance will also be analyzed (pre-time) (Fig. 1).

### Outcome parameters

**Fig. 2:**
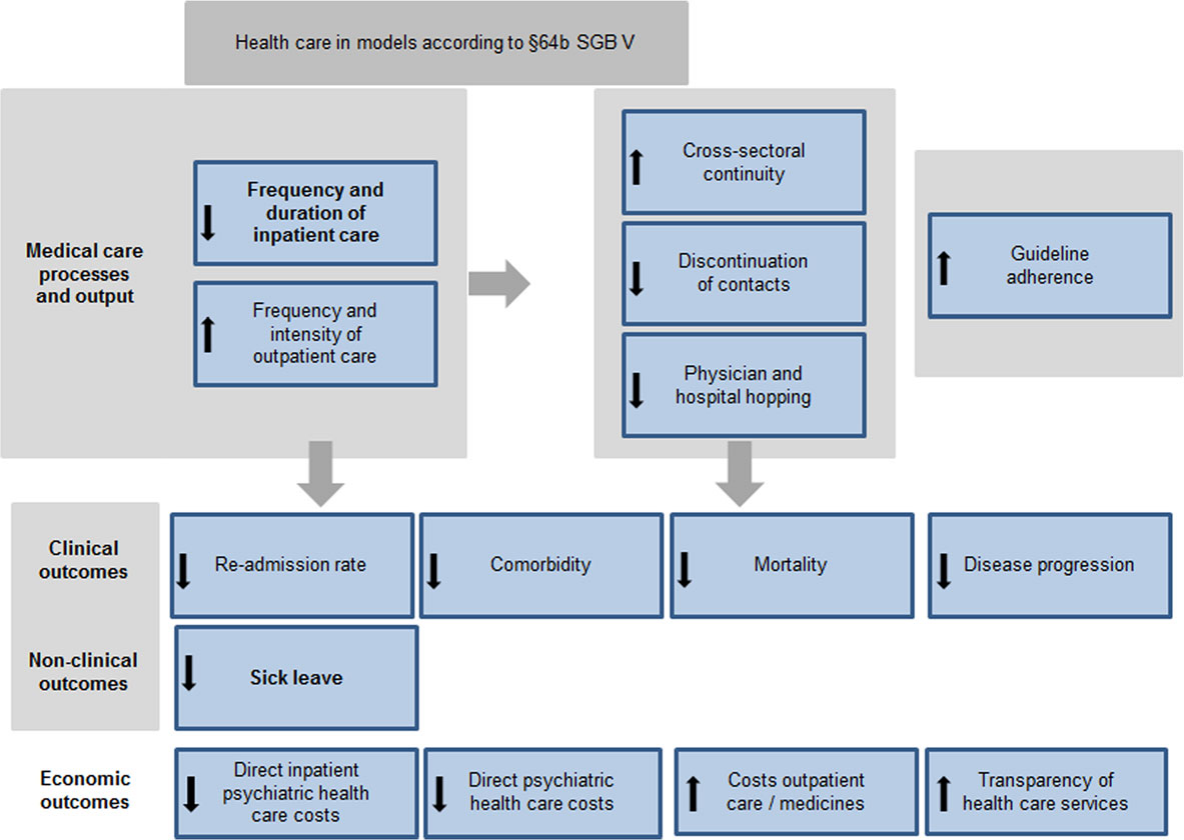
Outcome parameters and hypothesized effects, primary outcome parameters in bold printing

To operationalize the aims of the model projects, the following outcome parameters will be analyzed (Fig. 2):

### Primary outcome parameters

- frequency and duration of inpatient care, i.e. percentage of patients with inpatient care and length of hospital stay within 12 months after study inclusion,
- sick leave, i.e. percentage of patients with at least one sick leave episode, long-term sick leave (> 42 days in sick leave [33]) and number of days in sick leave within 12 months after study inclusion.

### Secondary outcome parameters

- frequency and intensity of outpatient care, i.e. number of outpatient contacts in the psychiatric health care system within 12 months after study inclusion,
- cross-sectoral continuity, i.e. percentage of patients having maximum 7, 30 or 90 days without outpatient contact to the psychiatric health care system after discharge from hospital within 12 months after study inclusion,
- discontinuation of contacts in psychiatric care, i.e. percentage of patients with severe mental disorder [34] without contact to the psychiatric health care system for more than 3 or 6 months after study inclusion [35],
- physician and hospital hopping, i.e. percentage of patients with more than two different health service providers within the same specialist department (inpatient) or the same group of specialist physician (outpatient) within 12 months after study inclusion,
- re-admission rate, i.e. percentage of patients with any psychiatric diagnosis in two hospital stays within 12 months after discharge from hospital [36, 37],
- comorbidity, i.e. number of comorbidities based on Elixhauser Score (ICD-10) without weighting [38],
- mortality, i.e. percentage of patients who die within 12 and 36 months after study inclusion,
- disease progression, i.e. percentage of patients who either go on from a mild/moderate depressive episode to a severe depressive episode, or from any depressive episode to a recurrent depressive disorder or who develop any addiction disorder within 12 months after study inclusion,
- guideline adherence, i.e. based on selected quality indicators for alcohol dependency, dementia, depression and schizophrenia which can be estimated using SHI data [39].

The outcome parameters cross-sectoral continuity, discontinuation of contacts in psychiatric care and re-admission rate will be stratified by whether the patient can be characterized as having severe mental disorder. Severe mental disorder is defined as.

(A) being diagnosed with any of the following disorders:

- F20.X-F22.X (schizophrenia),
- F25.X (schizo-effective disorders),
- F30.X (mania),
- F31.X (bipolar disorder),
- F32.2-F32.3 (severe depressive episode),
- F33.X (recurrent depressive disorder),
- F41.X (other anxiety disorders),
- F42.X (obsessive-compulsive disorder) or
- F60.31 (borderline personality) and as

(B) having any under (A) defined mental disorders diagnosed at least twice (two different quarters) each year during the last 2 years.

In addition, the following cost outcome parameters will be investigated, all secondary outcome parameters (Fig. 2):

- direct inpatient psychiatric health care costs,
- direct overall psychiatric health care costs,
- costs of outpatient care / medicines and transparency of health care services.

Costs as direct care costs within a period of 12 months after study inclusion are measured with SHI billing/accounting data, considering a differentiation of psychiatric vs. non-psychiatric care costs and health care sector where costs emerge.

Black arrows within the boxes in Fig. 2 symbolize the expected model effects, e.g. it is expected that the frequency and duration of inpatient care will decrease while the frequency and intensity of outpatient care will increase. Grey arrows between the boxes in Fig. 2 indicate the interdependencies between the outcome parameters.

All outcome parameters will be estimated for patients in model hospitals and compared to those of patients in control hospitals.

### Data

Information on 18 model projects will be evaluated. While the first included model projects started in January 2013, the latest model projects started in January 2017. Analyses will be based on health claims data provided by participating SHI funds. A common data set description was defined to determine the content and format of data extracted by the SHI funds. Data of inpatient and outpatient care including psychiatric outpatients department (PIA, for patients in need of particularly intensive and complex near-hospital care due to the nature, severity or duration of their mental disorder), of pharmaceutical and non-pharmaceutical treatments and of sick leave from 89 SHI funds will be analyzed. Once every year, all participating SHI funds send anonymized data on patients who fulfil inclusion criteria to the University of Magdeburg (data management unit of the study). The data transfer is based on a consensus of a data record description, which allows a homogenization of the 89 SHI funds data. The data management unit synthesizes, checks and merges the information from the different SHI funds and sends it to the TU Dresden and the WIG2 Leipzig for data analysis. While the TU Dresden evaluates model effectiveness, WIG2 analyzes cost information. Following this, TU Dresden and WIG2 jointly evaluate cost-effectiveness of models vs. routine care using cost-effectiveness analyses.

### Statistical analysis

All outcome parameters will be analyzed for the first year, the third year and the first 5 years (total evaluated model time) after the onset of the model project. The analysis for first or third year includes all patients that were initially treated in the model or control hospital during the first or third year of the contracts of the model hospital, respectively. The total evaluated model time includes all patients with initial treatment within the evaluation period, i.e. first 48 months after model start. Outcome parameters will be compared with the patient-individual pre-time (up to 2 years before study entrance). An a priori power analyses for the primary outcome parameters using α = 0.025 and 1 – β = 0.80 revealed that effects for even small hospitals could be verified with the estimated dataset.

Generalized multi-level models will be estimated controlling for dependencies within multiple observations within subjects. Furthermore, additional confounding regressors will be added. Regressors are mental disorders, severe mental disorders, age, sex, co-morbidities, care level, number of sick leave days before study inclusion, study inclusion through hospital or PIA. Covariance structures between these regressors will also be analyzed beforehand to identify potential multicollinearity [40]. Effects and costs will be analyzed from a SHI perspective. Primary (confirmatory) analyses will be performed according to the intention-to-treat approach. In the intention-to-treat approach, all patients will be evaluated as they will always be treated in the model or control hospital in which they were initially treated after start of the model project. Later changes in treatment hospitals will be disregarded.

In addition, the per-protocol approach is used to investigate the influence of drop-outs on the study results. In the per-protocol analysis, the following patients will be excluded from the analysis. Patients

- with at least two inpatient nights in a psychiatric hospital other than their allocated model or control hospital or
- with at least two visits in PIA of a psychiatric hospital other than their allocated model or control hospital or
- who were preponderantly treated in a psychiatric hospital other than their allocated model or control hospital.

A significance level of *p* ≤ 0.05 will be set for all analyses.

For the cost-effectiveness analysis, primary outcome parameters and costs will be compared between model and control hospitals using incremental cost-effectiveness ratio (ICER) as costs per one-day-of-hospital-stay avoided and as costs per one-day-of-sick-leave avoided. Cost-effectiveness planes will be used to visualize the bootstrapped replicates for the ICER [41, 42].

### Ethics and data protection

As exclusively anonymous data will be obtained from the statutory health insurance funds, the ethical committee of the University of Magdeburg confirmed that no ethical approval is necessary. Data are handled, analyzed and reported according to Good Epidemiological Practice (GEP) [43], Good Practice of Secondary Data Analysis (GPS) [26], a Consensus German Reporting Standard for Secondary Data Analyses, Version 2 (STROSA 2) [27] and German Recommendations on Health Economic Evaluation (Hanover Consensus) [44].

## Discussion

The evaluation of complex interventions in the health care sector is one main task of health service research and is the basis for evidence-based health care provision and development. Evaluations should investigate appropriate, structurally comparable control groups to avoid bias [45, 46]. One cornerstone of the described evaluation is the transparent and objective identification of appropriate control hospitals. As such an algorithm was not available before the start of this evaluation, the definition of this algorithm was one of the first steps of this evaluation. The algorithm has been shown to be practicable and expedient to identify appropriate control clinics for a priori defined model clinics based on administrative, especially claims data [32].

The merging and analysis of data from different statutory health insurance funds and the evaluation of complex interventions compared to routine care pose a huge technical and methodological challenge. Until today, there is no database in Germany encompassing all claims data of all SHI funds included in this study. This is why no other study in Germany has yet evaluated health claims data from so many different SHI funds. Prior experience about data handling and analysis is sparse. The experiences and findings from this study will generate methodological insight for further joint evaluations across different health insurance companies. Administrative data, especially claims data, offer opportunities to prospectively and retrospectively analyze detailed information where primary data are not available, impossible to retrieve or limited due to recall, information or further bias. While routine data offer essential information, preference-based and patient-centered information cannot be obtained. However, the presented methods are the best choice for use of administrative data to evaluate complex interventions.

The project EVA64 evaluates complex interventions regarding effectiveness, costs and efficiency of each model project separately as well as jointly to optimize the health care of patients with mental disorders in Germany. The evaluation on the basis of data from almost all German statutory health insurance funds allows a comprehensive evaluation of the health care of mental disorders. Such a common evaluation is unique in Germany and is, as indicated above, requested from other studies. A huge array of data including inpatient, outpatient, medication and sick leave will be used. In addition, the long-term evaluation allows for more stable evaluation and analysis. The described development of an objective selection of control hospitals can be used beyond this study. So far, no other study in Germany has analyzed such a large number of SHI funds. No other German project has brought together so many SHI funds for one common aim. No other evaluation project has yet evaluated so many model projects with one common study design and one database. Politics and SHI funds together have enabled researchers to form the basis of an evidence-guided decision on psychiatric care for adults and children/adolescents in Germany.

Information derived from this evaluation will give further insight into effectivity, cost and cost-effectivity of 18 model projects based on capitation principle. As requested by prior research, this project evaluates information about model projects on a common scientific basis [3, 24].

The indirect aim of the model projects is to evolve a system where the treatment can be adjusted flexibly to the patient and not the patient to the treatment. If the model projects in psychiatric care will be estimated to be efficient and cost-effective compared to routine care, this evaluation will provide arguments for a new structuring of routine care for patients with mental disorders in Germany.

## Abbreviations

ICD-10: International Classification of Disease, 10th revision
PIA: Psychiatric outpatients department
SGB: Social Security Code (Sozialgesetzbuch)
